# The Care of the Baby

**Published:** 1898-12

**Authors:** 


THE CARE OF THE BABY. By J. P. CROZER GRIFFITH, M. D., Clinical Professor of Diseases of Children, University of Pennsylvania. Second edition, revised. Duodecimo, 404 pages, with 67 illustrations in the text and 5 plates. Philadelphia: W. B. Saunders, 925 Walnut Street. 1898.

Dr. Griffith's book, the sub-title of which is a manual for mothers and nurses, is one of the best works of its kind that we have ever seen. The gieater part of the book is devoted to the hygiene of infancy; the baby's toilet, clothes, food, sleep, exercise and training being some of the topics discussed. This part of the book might be read with profit by the young physician, who is often lamentably ignorant of the details of the hygiene of early life. The sick baby receives attention at some length, the proper management of minor ailments being given, together with such instruction regarding the more serious forms of disease, as will serve to secure the intelligent coöperation of the mother when occasion arises. The appendix contains many useful recipes, directions for giving various baths, preparation of poultices, and the like.

